# Stability of gametocyte-specific *Pfs*25-mRNA in dried blood spots on filter paper subjected to different storage conditions

**DOI:** 10.1186/1475-2875-11-138

**Published:** 2012-04-30

**Authors:** Michael Pritsch, Andreas Wieser, Victor Soederstroem, David Poluda, Teferi Eshetu, Michael Hoelscher, Soeren Schubert, Jonathan Shock, Thomas Loescher, Nicole Berens-Riha

**Affiliations:** 1 Division of Infectious Diseases and Tropical Medicine, Medical Center of the University of Munich (LMU), Leopoldstrasse 5, 80802, Munich, Germany; 2 German Centre for Infection Research (DZIF) at LMU, Munich, Germany; 3 Max von Pettenkofer-Institute of Hygiene and Medical Microbiology, Munich, Germany; 4 Department of Microbiology, Parasitology and Immunology, Jimma University, Jimma, Ethiopia; 5 Max Planck Institute for Physics, Munich, Germany

**Keywords:** Malaria, Gametocytes, *Plasmodium falciparum*, *Pfs*25-mRNA, Dried blood spots, Filter paper, Real-time QT-NASBA, Storage conditions, Epidemiology

## Abstract

**Background:**

Real-time quantitative nucleic acid sequence-based amplification (QT-NASBA) is a sensitive method for detection of sub-microscopic gametocytaemia by measuring gametocyte-specific mRNA. Performing analysis on fresh whole blood samples is often not feasible in remote and resource-poor areas. Convenient methods for sample storage and transport are urgently needed.

**Methods:**

Real-time QT-NASBA was performed on whole blood spiked with a dilution series of purified *in-vitro* cultivated gametocytes. The blood was either freshly processed or spotted on filter papers. Gametocyte detection sensitivity for QT-NASBA was determined and controlled by microscopy. Dried blood spot (DBS) samples were subjected to five different storage conditions and the loss of sensitivity over time was investigated. A formula to approximate the loss of *Pfs*25-mRNA due to different storage conditions and time was developed.

**Results:**

*Pfs*25-mRNA was measured in time to positivity (TTP) and correlated well with the microscopic counts and the theoretical concentrations of the dilution series. TTP results constantly indicated higher amounts of RNA in filter paper samples extracted after 24 hours than in immediately extracted fresh blood. Among investigated storage conditions freezing at −20°C performed best with 98.7% of the *Pfs*25-mRNA still detectable at day 28 compared to fresh blood samples. After 92 days, the RNA detection rate was only slightly decreased to 92.9%. Samples stored at 37°C showed most decay with only 64.5% of *Pfs*25-mRNA detectable after one month. The calculated theoretical detection limit for 24 h-old DBS filter paper samples was 0.0095 (95% CI: 0.0025 to 0.0380) per μl.

**Conclusions:**

The results suggest that the application of DBS filter papers for quantification of *Plasmodium falciparum* gametocytes with real-time QT-NASBA is practical and recommendable. This method proved sensitive enough for detection of sub-microscopic densities even after prolonged storage. Decay rates can be predicted for different storage conditions as well as durations.

## Background

Mature gametocytes need to be present in human blood for transmission of *Plasmodium spp.* to anopheline vectors. Reliable prevalence data of gametocyte carriage in the population are needed to know the infectious reservoir and battle the ongoing transmission of malaria. This is especially important in the light of renewed malaria elimination and eradication efforts [1].

Gametocyte carriage alone does not cause clinical symptoms and gametocytes can remain in circulation for weeks after treatment. Microscopy can fail to detect gametocyte densities below 20 to 50 gametocytes/μl [2]. *In-vivo* and *in-vitro* feeding assays have shown, that even gametocyte densities below 1/μl can frequently result in mosquito infection. Although such low densities of circulating gametocytes reduce efficiency of malaria transmission [3], missing submicroscopic gametocytaemia represents a significant gap in mapping the infectious reservoir.

Modern molecular detection techniques of *Plasmodium* parasite nucleic acids like quantitative real-time PCR (qPCR) or QT-NASBA offer a limit of detection (LOD) down to 0.02 gametocytes per μl and thus can bridge this gap [4-6]. These amplification methods use gametocyte specific mRNA for quantification. Therefore, transport of fresh blood samples to a suitable laboratory has to be in a cool chain and samples need to be frozen until they are analysed in order to prevent significant and unpredictable loss of mRNA. However, areas heavily affected by malaria often do not offer sufficient infrastructure.

In order to facilitate sample collection and processing, dried blood spots (DBS) on filter paper can be used. This method does not provide results directly in the field [7], but increases the ability to store and transport large quantities of samples. Both, qPCR and QT-NASBA from DBS samples have been used for detection and quantification of *Plasmodium falciparum* gametocytes [8,9]. However, no investigation has been performed so far as to what extent storage conditions affect detection. This study was designed to create settings which could realistically be encountered in resource-poor conditions. Assays were standardised with *in-vitro* gametocyte cultures spiked in whole blood. QT-NASBA was performed on fresh whole blood (FWB) samples as well as DBS filter paper samples stored under different conditions. The results were compared in detail and recovery and decay rates for all storage conditions were calculated. *In-vivo* samples consisted of DBS filter papers as well as thick blood films collected from malaria patients in Jimma, Ethiopia.

## Methods

Gametocytes for the dilution series were obtained from a continuous *in-vitro* parasite culture of *Plasmodium falciparum* (strain NF 54, Amsterdam) synchronised with sorbitol [10]. The parasite density of the cultures ranged from 2-10% infected erythrocytes. Culture was grown in sterile filtered medium (RPMI 1640 supplemented with 25 mM HEPES, 25 mM sodium bicarbonate, 25 mg/l gentamicin sulphate, 2% D-glucose, and 10% human type AB serum, pH 7.4) with additional erythrocytes to uphold a haematocrit of 5%. New medium to feed the culture was added twice daily. The cultures were kept under a gas phase of 3% O_2_, 5% CO_2_ and 92% N_2_[11,12].

Cultures were harvested at a parasitaemia of approximately 10%, pooled until 100 ml of total volume was reached and centrifuged at 1,700 g for 10 minutes. The resulting pellet was resuspended in 10 ml prewarmed pure RPMI medium. To increase the density and to concentrate gametocytes, the diluted pellet was filtered through MACS® Separation Columns (Miltenyi Biotec, Bergisch Gladbach, Germany) as described in more detail by Ribaut and colleagues [13]. This resulted in 250 μl of enriched solution which was analysed for gametocyte density. Gametocyte counts were independently performed by three trained microscopists using an oil immersion microscope (Axiolab, Carl Zeiss MicroImaging GmbH, Göttingen, Germany). The enumeration was based on three 1 μl Giemsa-stained thick films. The total surface area of each individual spot was measured and 20% thereof was counted. The mean gametocyte density was calculated based on these data.

To investigate the quantification accuracy and the LOD of real-time QT-NASBA, a 10-fold dilution series ranging from 2.5 × 10^-3^ to 2.5 × 10^3^ gametocytes per μl was prepared in type AB fresh blood. The calculated mean gametocyte density from each dilution was used for further calculations. To microscopically control the dilution process, three 1 μl Giemsa-stained thick films were prepared for densities of 2.5 × 10^0^ to 2.5 × 10^2^ gametocytes per μl. All these thick films were entirely counted and the mean density was determined. Densities below 10^0^ per μl were not considered evaluable by microscopy.

Dried blood spots on filter paper (Standard-Whatman Cellulose Chromatography paper 3MM, GE Healthcare, Fairfield, CT, USA) were prepared from 50 μl of the whole blood dilution series, dried for three hours at room temperature, labelled and individually sealed in plastic bags. Samples were then subjected to different storage conditions (as outlined below).

### Storage procedures

The DBS filter papers of each dilution series were analysed after 24 hours, 28 days and 92 days. The processing involved nucleic acid extraction, amplification and quantification with QT-NASBA. To investigate the effect of different storage procedures, the filter papers were subjected to the following conditions:

(I): Transport simulation (+5-30°C, alternating relative humidity (RH))

(II): Room temperature (+25°C, 38% RH)

(III): Warm storage (+37°C, 25% RH)

(IV): Fridge (+8°C, 74% RH)

(V): Freezer (−20°C, 61% RH)

### RNA extraction

Right before analysis, the DBS containing 50 μl of blood were cut out of the filter paper with a distance of approximately 3 mm to the blood spot using heat sterilised scissors. The blood spots were immediately soaked in 2 ml NucliSens® easyMAG® (bioMérieux, Lyon, France) lysis buffer containing (guanidiumisothiocyanate, GuSCN) and rocked at 150 rpm for 30 minutes at room temperature. The solution was subsequently centrifuged at 1,500 g for 5 minutes and the filter paper was removed. Fresh whole blood was treated according to the manufacturer’s instructions. After addition of 50 μl of silica particle solution, the original RNA extraction method described by Boom et al. [14] was performed. RNA was extracted with the NucliSens® - miniMAG™ unit (bioMérieux, Lyon, France) according to the manufacturer´s instructions. Nucleic acids were eluted in 30 μl elution buffer as supplied by the manufacturer.

After the extraction, the specimen was either immediately amplified using NASBA technology or stored at −80°C for up to 24 hours for further analysis. Filter papers spotted with 50 μl of plasmodium-negative full blood were used as negative controls for all steps of analysis.

### Real-time quantitative nucleic acid sequence-based amplification (QT-NASBA)

The *Pfs*25-mRNA was chosen as target sequence as it is expressed only in *P. falciparum* stage V gametocytes [15]. Primers *Pfs*25.F (5‘-GACTGTAAATAAACCATGTGGAGA-3) and *Pfs*25.R (5‘-T7-CATTTACCGTTACCACAAGTTA-3‘) and the molecular beacon probe (5‘-TexasRed-CGATCGCCCGTTTCATACGCTTGTAA-CGATCG-DABSYL-3‘) were used at a final concentration of 290 nM and 145 nM, respectively, as described elsewhere [15-17]. The amplification was performed using the NucliSENS EasyQ® Basic Kit (bioMérieux, Lyon, France) according to the manufacturer’s instructions at a KCl concentration of 80 mM. In brief, 5 μl of RNA eluate was incubated at 65°C for 2 min and subsequently at 41°C for 2 min together with 10 μl reaction mixture including primers and beacons. Prior to isothermal amplification at 41°C, an enzyme mixture containing AMV-RT, RNAse H and T7 RNA polymerase was added to a total reaction volume of 20 μl. Real-time amplification was allowed to run for 90 minutes.

Samples were considered positive when the time-point of amplification at which the fluorescence detecting target amplicons (time to positivity = TTP) exceeded the mean fluorescence of three negative controls + 20 SD as described by Schneider *et al*[6].

### Data analysis

The raw data from the QT-NASBA reactions were analysed with Mathematica 8.0 (Wolfram Research, Champaign, IL, USA) and Sigma Stat (systat Software GmbH, San José, CA, USA) software. Student’s t-test was used for significance testing of normally distributed data sets. Otherwise, Whitney-Man rank sum test was performed. P-values of <0.05 were considered statistically significant. All P-values are indicated within the figures or the text as appropriate.

## Results

### Limit of detection

A 1:10 dilution series with seven concentrations of descending gametocyte densities between 2.5 × 10^3^ – 2.5 × 10^-3^/μl was prepared. Real-time QT-NASBA performed from extracted fresh whole blood spiked with gametocyte culture showed highly significant correlations with theoretical dilution densities (r^2^ = 0,883, p = <0,001) of the samples as well as with microscopic results (r^2^ = 0.878, p = <0,001). In the correlations, there was a slight trend towards higher values of the theoretical dilution compared to microscopy and also between NASBA and microscopy. More than 3 samples were tested at each density for each storage method at every time except for day 1, which was mainly tested in duplicates, but repeatedly. Quintiplicate samples for each density were tested at day 0. Decay rates were individually calculated for all different storage conditions. Decay of mRNA was measured indirectly by increases in TTP of NASBA, as no method for direct enumeration of *Pfs*25-mRNA exists.

**Figure 1:**
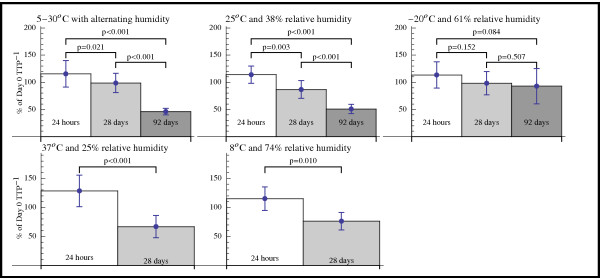
**Rates of*Pfs*25-mRNA retrieved from DBS filter paper samples subjected to different storage conditions.** Analysis was performed from extracted DBS filter papers and standardised against fresh whole blood at the time of sample preparation (100%). QT-NASBA-1/TTP was used as a surrogate parameter for the amount of mRNA. Depicted retrieval rates for each time point are averaged over all gametocyte densities (10^-1^ to 10^3^ per μl) and plotted in a single column. Statistical significance and storage conditions are labelled above the individual charts.

Real-time QT-NASBA results from extracted DBS aged 24 h yielded similar results when correlated with theoretical dilution densities as well as microscopy (r^2^ = 0,754, p = <0,001 and r^2^ = 0,747, p = <0,001 respectively). TTP was significantly shorter for DBS aged 24 h than for fresh whole blood (Figure 1, p = 0.002). Similar results were obtained in a related study using gametocyte-spiked cell free medium (unpublished observation).

After 28 days, significantly lower amounts of *Pfs*25-mRNA could be detected in almost all DBS filter papers subjected to the different storage conditions (Figure 1). Storage in the freezer (−20°C) resulted in slower decay of specific mRNA, the trend was not significant (p = 0.152). In comparison with day 0, the mean amount of detected gametocytes in the filter papers of all storage conditions after 28 days was 85.4% (SD: 13.9). However, as expected the different storage procedures caused varying loss of detectable *Pfs*25-mRNA (Figure 1).

After 92 days, QT-NASBA was performed on extracted samples to evaluate the influence of temperature, movement and/or alternating humidity on retrieval of *Pfs*25-mRNA. Samples stored in the freezer were found to decay the least. The mRNA levels exhibit a downward trend from day 1 to day 92, however the loss was not significant between day 1 and day 28 (p = 0.152), day 28 and day 92 (p = 0.353) as well as between day 92 and day 1 (p = 0.111).

**Figure 2:**
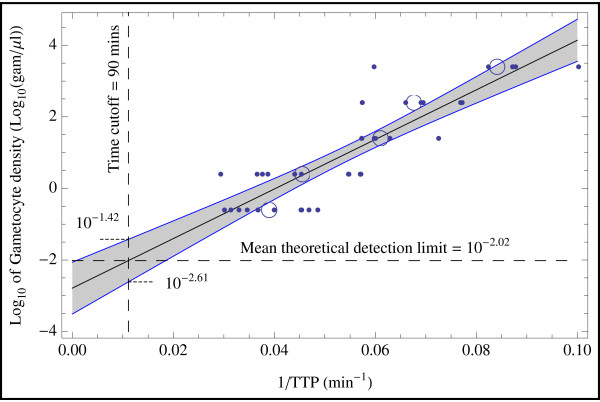
**Linear regression analysis for determination of the theoretical gametocyte detection limit in 24 h-old DBS samples.** QT-NASBA-1/TTP plotted against the log of gametocyte concentration per μl for each 24 h DBS filter paper sample duplicate. Dots represent the raw data and circles represent the average over a particular density. All storage conditions were summarised, as TTP-levels did not differ significantly. Linear regression analysis was performed based on all data points. The longest TTP was set to 90 minutes, indicated by the dashed vertical line. The mean theoretical limit of detection at 90 minutes (0.0095) was calculated and is indicated by the horizontal dashed line. The 95% confidence interval of the regression analysis was calculated with Scheffé’s method (0.0025 to 0.0380) per μl.

In order to calculate the theoretical LOD (the minimum density for which gametocytes could theoretically still be detected) the 24 h-old filter paper samples were used. All storage procedures were combined in a single analysis, as they did not show any significant differences in mRNA-retrieval and provided more data points for accurate analysis. The theoretical LOD of 0.0095 (95% CI: 0.0025 to 0.0380) gametocytes per μl was calculated with linear regression analysis and Scheffé’s method (Figure 2). It theoretically permits the detection of a single gametocyte in each blood spot sample (50 μl). However, due to dilution and sampling artefacts that have to be expected with low concentrations, a practical LOD should be simulated. Monte Carlo simulation was performed to determine the minimal concentration providing 95% confidence of gametocyte detection for a 50 μl DBS. Assuming homogenous distribution of gametocytes in the blood stream, the practical LOD was expected to be 0.065/μl, corresponding to 3.25 gametocytes per 50 μl blood spot.

### Decay rates

To approximate the loss of mRNA due to a sequence of different storage procedures, this mathematical formula was derived:

(1) $$\begin{array}{l} \%\;\text{ of }\;1/\text{TTP compared to day }\;0=\left(118\;\pm \;8.9\right)\;{\text{e}}^{\text{SIdI}+\text{SIIdII}+\text{SIIIdIII}+\text{SIVdIV}+\text{SVdV}}\\ {\text{S}}_{\text{I}}=-0.00976495\pm 0.00113332\\ {\text{S}}_{\text{II}}=-0.00892571\pm 0.000862804\\ {\text{S}}_{\text{III}}=-0.0241597\pm 0.00402791\\ {\text{S}}_{\text{IV}}=-0.0152481\pm 0.00300704\\ {\text{S}}_{\text{V}}=-0.00180098\pm 0.00126801 \end{array}$$

The variable marked with “d” represents the time measured in days the sample was stored under conditions I to V. S_I_ - S_V_ indicate the individual decay rates as listed above. The correction factor (118 ± 8.9) represents the increase in % of 1/TTP experienced when analysing dried blood spots on filter paper in comparison to whole fresh blood samples.

An example for the calculation of estimated loss of mRNA for a sequence of different storage procedures can be found in Additional file 1.

The filter paper samples consistently revealed higher results for 1/TTP after one day of storage in comparison to the current gold standard of fresh whole blood. In order to rule out a responsibility of single-stranded DNA for this effect, extracted samples from 50 μl DBS as well as 50 μl fresh whole blood spiked with a gametocyte density of 0.28 gametocytes/μl were subjected to DNAse and RNAse treatment. After 15 min digestion at 37°C, the enzymes were heat inactivated at 75°C for 20 min according to the manufacturer's protocol. The control extractions were equally treated but without any enzymes. It could be demonstrated that the samples were reduced to the level of the negative control after RNAse treatment whereas the DNAse treatment did not alter the level of detected mRNA significantly (Additional file 2).

To investigate whether distinct makes of filter paper influence the correction factor, the four different types Standard-Whatman Cellulose Chromatography paper 3MM, MFTA-Whatman FTA Elute Micro card, 903-Whatman 903 Protein Saver card and FTA–Whatman FTA Classic card were compared with each other and to freshly extracted whole blood. The gametocyte density of the blood samples used was 2.25 gametocytes/μl. No difference between the four tested filter papers could be observed and the correction factor was found to be stable (Additional file 3).

## Discussion

This report evaluates the stability of gametocyte-specific *Pfs*25-mRNA in dried blood spots on filter paper subjected to different storage conditions. Two methods are widely used to quantify gametocytes in different materials: QT-NASBA and qPCR. There are several advantages of QT-NASBA amplification over qPCR. Primarily, the reverse transcription step to produce complementary DNA (cDNA) is dispensable resulting in faster sample processing and less opportunities to lose RNA or to contaminate samples. As isothermic amplification does not require cycles, amplification time can be reduced [6,15,18]. If QT-NASBA could be performed without extraction, this would further speed up sample preparation [7]. The primer attachment sites for qPCR and QT-NASBA can be chosen to be at similar positions within the coding sequence. However, as many genes in *Plasmodium* do not contain introns, potential cDNA is identical to genomic sequences. Hence, qPCR is totally dependent on complete removal of genomic DNA in contrast to QT-NASBA [15]. In summary, real-time QT-NASBA seems to be more convenient and faster for gametocyte quantification.

Dried blood on filter paper has already been used effectively for gametocyte detection by amplifying specific mRNA [9]. In order to implement this approach in large-scale studies, the estimation of the original gametocyte density should preferably be feasible any time after sample collection - at least up to 3 months afterwards.

No significant differences in detectable mRNA levels for different storage procedures within the first 24 hours were observed. Apparently, temperatures in the range of −20 to 37°C seem to be of little importance for short-term storage as long as the samples have been handled and dried appropriately. Surprisingly, in all DBS subjected to different storage conditions for only 24 hours, significantly more *Pfs*25-mRNA was detected than in freshly extracted whole blood samples (p = 0.002).

To exclude interference with single-stranded DNA (ssDNA), DNAse- and RNAse- treated samples were amplified. Results indicated no influence of ssDNA. Moreover, it was hypothesised that different makes of filter paper might interfere with the process. So far, the outcome seemed not to be affected by use of four different kinds of filter papers stored for 24 hours at room temperature.

To investigate if fresh whole blood interferes with kit reagents by i.e. inhibiting the extraction or amplification reaction, the whole study was also repeated with gametocyte-spiked whole medium producing similar results. Perhaps the drying process on filter paper might facilitate the lysis of parasites by damaging their walls, resulting in a more efficient lysis than in fresh blood.

By contrast, investigations performed with HIV-1-RNA on DBS demonstrated a significant loss of RNA during the drying process and within the first hours after preparation of filter paper samples compared to fresh whole blood [19,20]. Perhaps HIV-1-RNA is more accessible in the beginning or degrades faster during the drying process.

In conclusion, the correction factor for signal increase was found to be stable in the specific study settings and was not influenced by the tested storage procedures. It is however recommended to determine the individual correction factor depending on the workflow used for each study.

After 28 days, significant differences between the storage conditions could be observed. As expected, the freezer (−20°C) prevented most efficiently RNA decay (average recovery rate 98.7%) and 37°C performed worst (average recovery rate 66.7%) (see also Figure 1). Similar results could be obtained after 92 days of storage. At day 28, storage in the fridge showed a slight, but insignificant, trend towards higher decay rates. This trend was only found once in the experiment selected for presentation and not in the other repetitions. It can be assumed that this trend was due to statistical variation. The experiment was neverthless selected as it was the most extensive performed with storage times of up to 92 days (transport simulation, room temperature and freezer).

Exponential decay rates for each storage condition were calculated, allowing estimates of signal loss experienced for each individual sample as long as storage conditions and duration were known. Naturally, all calculations rely on standardised procedures and might vary depending on the individual setting.

The theoretical limit of detection (LOD) was calculated by linear regression with over 42 duplicates of 24 h-old DBS. A density of less than 10 gametocytes per ml could be detected (0.0096/μl, 95% CI 0.0025 to 0.038). Nevertheless, due to methodical non-linearity of the QT-NASBA reaction for target concentrations with absolute extremes, the linearity of regression is only an approximation. LODs close to one gametocyte per DBS are subject to huge dilution effects and stochastic noise. In addition, the LOD will decrease with prolonged storage due to target decay. However, an individual reasonable LOD can be estimated with the data presented above.

QT-NASBA amplification of DBS was performed from Ethiopian patients, who were tested negative for gametocytes by microscopy in 2008 and 2009. Interestingly, 22 (69%) of 32 cases were detected positive for gametocytes nevertheless with QT-NASBA (Additional file 4). These results were astonishing, as the samples had been stored for 2–12 months at room temperature and subsequently 18–30 months at −20°C. Significant decay had to be expected. However, successful RNA extraction has also been described after long-term storage if the initial load was high enough [21].

Although it is unlikely that a mosquito blood meal of 2–3 μl at gametocyte densities below 0.20-0.30 gametocytes per μl contains at least one male and one female gametocyte required for fertilisation [2], Schneider and colleagues could prove that some patients tested negative for *Pfs*25-mRNA with QT-NASBA could still infect mosquitoes [16]. It is yet to be investigated to what extent these very low-level gametocyte densities contribute to the infectious reservoir and at what frequency they result in mosquito infection. Sub-microscopic gametocyte carriage over longer time scales is known to play an important role in sustaining transmission [22].

## Conclusions

The results of this study suggest that DBS filter papers constitute a practical and cost-effective blood sample transport and storage method for studies on gametocyte carriage. Real-time QT-NASBA performed of DBS results in accurate and sensitive detection of submicroscopic gametocyte levels. By investigating different common storage procedures over time, individual decay rates could be calculated. This allows an estimation of the original gametocyte count for each sample as long as the blood volume and storage conditions are known. DBS filter papers represent a practicable tool for epidemiological studies and clinical trials with anti-malarial drugs or vaccines.

## Competing interests

The authors declare that they have no competing interests.

## Authors’ contributions

VS, MP, NBR, MH, SS and AW designed the experiments. MP, VS, DP and NBR performed real-time QT-NASBA and cultured parasites. MP, TE and NBR performed microscopy. TE, NBR and LT provided clinical samples. JS, MP, VS, AW and NBR performed data analysis. MP, VS, DP, NBR, JS and AW wrote the manuscript. All authors read and approved the final manuscript.

## Supplementary Material

Additional file 1 **Calculated retrieval rates of*Pfs*25-mRNA in a hypothetical storage scenario.** The measured decay rates for individual storage procedures were used to derive a formula, which simulates retrieval rates of *Pfs*25-mRNA after certain time periods. The figure presents the storage simulation using a backpack (one week), a fridge (one month) and finally a freezer (two months). Ultimately an average recovery rate of 48.0% would be achieved. Click here for file

Additional file 2 **Comparison of DNAse and RNAse treated samples from a 24 h old extracted DBS stored at room temperature.** It is known that ssDNA can be amplified in an RNA-based NASBA setting [14,15], therefore additional experiments were performed. Standard filter paper (Whatman Chromatography 3MM) spotted with a 50 μl blood sample of 0.28 gametocytes/μl (average of 14 gametocytes in the whole spot) was extracted. The eluate of nucleic acid extraction was then separated into three portions. One was treated with DNAse (DNAse I,1U/μl, Fermentas) and the other with RNAse (RNAse A, 10 mg/ml, Fermentas) respectively. The third sample was stored next to the treated tubes in the shaker and heat block and served as a control. The enzymatic treatment enables a direct comparison of amplification from DNA and RNA free samples. Incubations at 37°C for 15 minutes followed by 75°C for 20 minutes to inactivate the enzymes was required. As expected, the heat treatment reduced the signal amplitude slightly compared to other control samples incubated on ice, probably due to nucleic acid decay during incubation at the higher temperatures. However, it could be demonstrated, that the time to positivity measured by QT-NASBA did not differ between the DNAse treated samples and the heat treated control and no signal could be acquired from the RNAse treated samples. This data excludes the presence of plasmodium ssDNA or dsDNA as cause for the signal increase experienced after 24 h from filter paper. The same experiment was also performed with extracted whole blood dilutions and confirmed the results. Click here for file

Additional file 3 **Comparison of different types of filter papers in the ability for Pfs25-mRNA retrieval.** In order to evaluate how different additives and qualities of filter papers actually affect the stability of RNA, standard Whatman Chromatography paper without impregnation (3MM Chr, Catalog number 3030 917) was used as a reference compared to the FTA classic cards (catalog number WB120205) [Tsumori et al 2011], FTA Micro Card (catalog number WB120210) and the 903 Protein Saver Card (catalog number 10534612) [Shekalaghe et al 2011]. Filter papers were spotted with 50 μl fresh whole blood containing 2.25 gametocytes/μl, dried and kept in sealed plastic bags at room temperature. RNA extraction and immediate NASBA amplification after 24 h of storage did not show any differences between the different makes. Click here for file

Additional file 4 Clinical DBS samples Click here for file
