# Supplementary figures and images for: Stability of gametocyte-specific Pfs25-mRNA in dried blood spots on filter paper subjected to different storage conditions
Source: Malar J. 2012 Apr 30;11:138. doi: 10.1186/1475-2875-11-138 (PMC3778852; doi:10.1186/1475-2875-11-138)

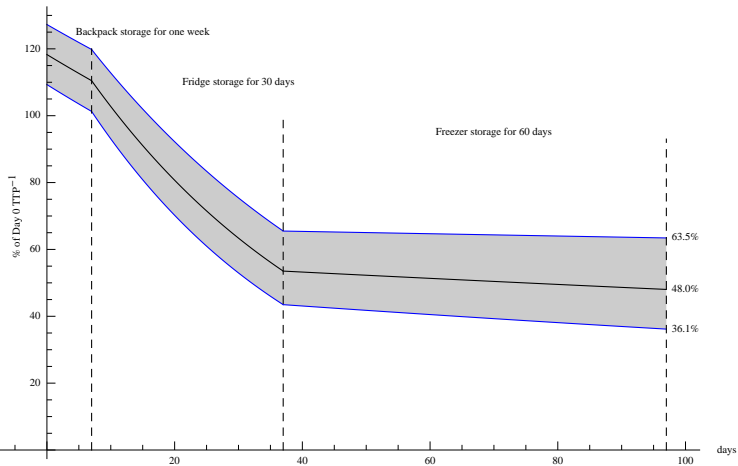

Supplement: Additional file 1 — Calculated retrieval rates ofPfs25-mRNA in a hypothetical storage scenario. The measured decay rates for individual storage procedures were used to derive a formula, which simulates retrieval rates of Pfs25-mRNA after certain time periods. The figure presents the storage simulation using a backpack (one week), a fridge (one month) and finally a freezer (two months). Ultimately an average recovery rate of 48.0% would be achieved. [file 1475-2875-11-138-S1.pdf]

## Fresh whole blood

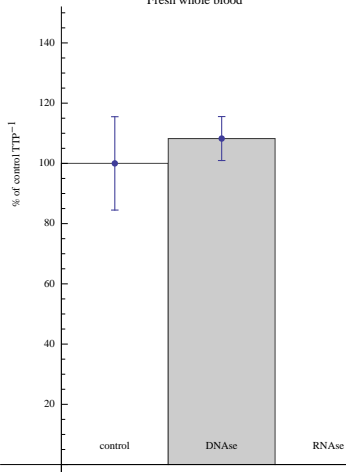

## Room temperature day 1

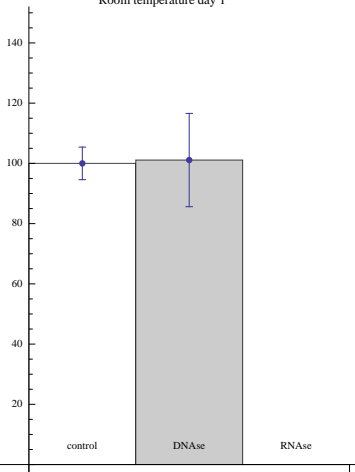

Supplement: Additional file 2 — Comparison of DNAse and RNAse treated samples from a 24 h old extracted DBS stored at room temperature. It is known that ssDNA can be amplified in an RNA-based NASBA setting [14,15], therefore additional experiments were performed. Standard filter paper (Whatman Chromatography 3MM) spotted with a 50 μl blood sample of 0.28 gametocytes/μl (average of 14 gametocytes in the whole spot) was extracted. The eluate of nucleic acid extraction was then separated into three portions. One was treated with DNAse (DNAse I,1U/μl, Fermentas) and the other with RNAse (RNAse A, 10 mg/ml, Fermentas) respectively. The third sample was stored next to the treated tubes in the shaker and heat block and served as a control. The enzymatic treatment enables a direct comparison of amplification from DNA and RNA free samples. Incubations at 37°C for 15 minutes followed by 75°C for 20 minutes to inactivate the enzymes was required. As expected, the heat treatment reduced the signal amplitude slightly compared to other control samples incubated on ice, probably due to nucleic acid decay during incubation at the higher temperatures. However, it could be demonstrated, that the time to positivity measured by QT-NASBA did not differ between the DNAse treated samples and the heat treated control and no signal could be acquired from the RNAse treated samples. This data excludes the presence of plasmodium ssDNA or dsDNA as cause for the signal increase experienced after 24 h from filter paper. The same experiment was also performed with extracted whole blood dilutions and confirmed the results. [file 1475-2875-11-138-S2.pdf]

filter paper day 1 – room temperature

density 2.25 G/ $\mu$ l

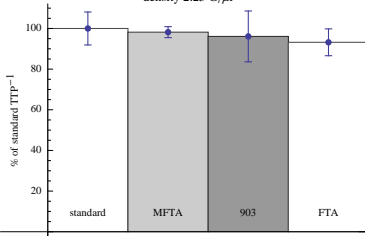

density 0.281 G/ $\mu$ l

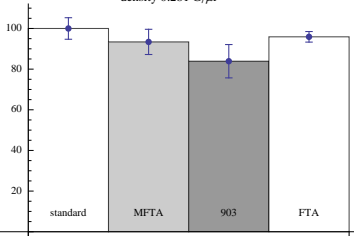

Supplement: Additional file 3 — Comparison of different types of filter papers in the ability for Pfs25-mRNA retrieval. In order to evaluate how different additives and qualities of filter papers actually affect the stability of RNA, standard Whatman Chromatography paper without impregnation (3MM Chr, Catalog number 3030 917) was used as a reference compared to the FTA classic cards (catalog number WB120205) [Tsumori et al 2011], FTA Micro Card (catalog number WB120210) and the 903 Protein Saver Card (catalog number 10534612) [Shekalaghe et al 2011]. Filter papers were spotted with 50 μl fresh whole blood containing 2.25 gametocytes/μl, dried and kept in sealed plastic bags at room temperature. RNA extraction and immediate NASBA amplification after 24 h of storage did not show any differences between the different makes. [file 1475-2875-11-138-S3.pdf]
