# Supplementary material for: Stability of gametocyte-specific Pfs25-mRNA in dried blood spots on filter paper subjected to different storage conditions
Source: Malar J. 2012 Apr 30;11:138. doi: 10.1186/1475-2875-11-138 (PMC3778852; doi:10.1186/1475-2875-11-138)
Supplement: Additional file 4 — Clinical DBS samples [file 1475-2875-11-138-S4.pdf]

Additional file IV: Clinical DBS samples

60 random finger prick DBS filter paper and thick film samples of malaria patients in the region of Jimma, Ethiopia, were chosen for the analysis. The samples were collected between 2008 and 2009 at Jimma University Hospital. The finger prick DBS were approximated to consist of a sample volume of 10 µl. However, no standardised collection technique was used at that time. The samples had been stored for 2-12 months at room temperature and subsequently 18-30 months at -20°C until RNA extraction and QT-NASBA amplification was performed. Parasite density was microscopically counted based on standard procedures. In brief, the number of asexual parasites was counted against 200 leukocytes assuming 8,000 leukocytes per µl. Smears were determined to be negative only after examining 120 fields [100x ocular]. Differences in parasite densities of more than 15% between microscopists were checked by a third microscopist, whose counting was final. 28 (46.7%) were microscopically positive for gametocytes, the other 32 samples were microscopically negative. Gametocyte *Pfs*25-mRNA was detected in 43 (71.7%) of all samples. The correlation between the microscopically investigated gametocyte density of the DBS filter paper samples and the QT-NASBA *Pfs*25-mRNA was impossible to calculate due to the unknown extracted blood volume. A 7 out of 28 microscopically positive samples were negative by QT-NASBA and 22 of 32 microscopically negative samples were positive by QT-NASBA. There were 31 concordant pairs. The McNemar's chi-squared test for paired samples showed strong evidence for a real difference between the two tests ($p < 0.01$). There was no statistically significant difference between samples collected in 2008 or in 2009.
